# Medication-Wide Association Study Using Electronic Health Record Data of Prescription Medication Exposure and Multifetal Pregnancies: Retrospective Study

**DOI:** 10.2196/32229

**Published:** 2022-06-07

**Authors:** Lena Davidson, Silvia P Canelón, Mary Regina Boland

**Affiliations:** 1 Biostatistics, Epidemiology & Informatics University of Pennsylvania Philadelphia, PA United States

**Keywords:** pregnancy, pregnancy, multiple, assisted reproductive technique, electronic health record

## Abstract

**Background:**

Medication-wide association studies (MWAS) have been applied to assess the risk of individual prescription use and a wide range of health outcomes, including cancer, acute myocardial infarction, acute liver failure, acute renal failure, and upper gastrointestinal ulcers. Current literature on the use of preconception and periconception medication and its association with the risk of multiple gestation pregnancies (eg, monozygotic and dizygotic) is largely based on assisted reproductive technology (ART) cohorts. However, among non-ART pregnancies, it is unknown whether other medications increase the risk of multifetal pregnancies.

**Objective:**

This study aimed to investigate the risk of multiple gestational births (eg*, twins* and *triplets*) following preconception and periconception exposure to prescription medications in patients who delivered at Penn Medicine*.*

**Methods:**

We used electronic health record data between 2010 and 2017 on patients who delivered babies at Penn Medicine, a health care system in the Greater Philadelphia area. We explored 3 logistic regression models: model 1 (no adjustment); model 2 (adjustment for maternal age); and model 3—our final logistic regression model (adjustment for maternal age, ART use, and infertility diagnosis). In all models, multiple births (MBs) were our outcome of interest (binary outcome), and each medication was assessed separately as a binary variable. To assess our MWAS model performance, we defined ART medications as our gold standard, given that these medications are known to increase the risk of MB.

**Results:**

Of the 63,334 distinct deliveries in our cohort, only 1877 pregnancies (2.96%) were prescribed any medication during the preconception and first trimester period. Of the 123 medications prescribed, we found 26 (21.1%) medications associated with MB (using nominal *P* values) and 10 (8.1%) medications associated with MB (using Bonferroni adjustment) in fully adjusted model 3. We found that our model 3 algorithm had an accuracy of 85% (using nominal *P* values) and 89% (using Bonferroni-adjusted *P* values).

**Conclusions:**

Our work demonstrates the opportunities in applying the MWAS approach with electronic health record data to explore associations between preconception and periconception medication exposure and the risk of MB while identifying novel candidate medications for further study. Overall, we found 3 novel medications linked with MB that could be explored in further work; this demonstrates the potential of our method to be used for hypothesis generation.

## Introduction

Multifetal pregnancies are at a high risk for obstetric complications, including anemia, preterm labor, pregnancy-induced hypertension, placental previa, and fetal malformations [1,2]. These pregnancies pose a risk of adverse fetal and infant outcomes and an increased risk of maternal morbidity and mortality [3,4]. Multifetal pregnancy can occur due to genetic and environmental factors, with higher maternal age, advanced parity, and use of assisted reproductive technology (ART) as established factors in multifetal pregnancy [5]. Although the etiology of dizygotic twins is in most cases straightforward (eg, increase in the number of embryo transfers and medications that increase oocyte release), the etiology of increased monozygotic twinning is less well characterized outside of ART use and fertility treatments [6].

ART is a widely accepted treatment for infertile couples, referring to all treatments that include the handling of eggs, sperm, and embryos. Outside the scope of ART, hormonal medications for the purpose of facilitating a successful pregnancy are referred to as fertility treatment. Increased rates of monozygotic twinning have been observed in pregnancies due to ART use (ie, in vitro fertilization [IVF], micromanipulation, multiple embryo transfer, and gonadotrophin treatment) [6-8]. Ovulation induction (eg, gonadotrophin treatment) therapy may predispose to monozygotic twinning or greater survival of monozygotic twins after their formation [6]. An estimated 1.8% of births in the United States in 2016 were conceived with ART, of which approximately 30.4% were twins and 1.1% were triplets. In animal models, mitotic inhibitors and teratogenic agents were observed to induce monozygotic twinning [9]. In humans, the mechanism of induction of spontaneous twinning remains unknown; twinning-inducing factors outside of ART are thought to involve an environmental exposure (eg, medications and teratogenic agents) during a critical window of pregnancy [9].

The wealth of information from electronic health record (EHR) data can allow for hypothesis-driven research on the associations between medications and pregnancy outcomes. Ryan et al [10] proposed a medication-wide association study (MWAS) approach, in which an outcome of interest is compared with all drugs available for comparison. This approach has been applied to a variety of health outcomes, including cancer risk [11,12]; spontaneous preterm birth [13]; acute myocardial infarction [10,14,15]; and acute liver failure, acute renal failure, and upper gastrointestinal ulcers [10].

Except for research conducted on nationwide health care data registries [11,12,14], MWAS approaches often depend on administrative claims data [10,13,15]. We aimed to present a methodology to systematically explore potential associations between the medications prescribed during the preconception and first trimester period and the occurrence of multiple birth (MB) in patients who delivered at Penn Medicine. Existing screening tools for multifetal pregnancies aim to characterize perinatal morbidity and mortality [16,17], observe noninvasive prenatal screening techniques [18], detect twin-twin transfusion syndrome [19], determine intertwin weight discordance [20], predict MB risk [21], and discover other associations with pregnancy complications [22]. A multitude of these studies depend on IVF clinical data [21], involve increased fetal monitoring [19,20,22], concern twin pregnancy management [18], or are focused on pregnancy complications associated with MB [16,17,22]. Our literature review found no prior research that observes prescription medication use during the periconceptual and first trimester period and its association with MB, let alone using EHR data.

This study illustrates a proof-of-concept MWAS approach for hypothesis-driven pharmacovigilance research on EHR data, with a particular focus on MB.

## Methods

### Data Source and Identification of MB (Outcome)

We used EHR data obtained from 4 different hospitals within the Penn Medicine system: the Hospital of the University of Pennsylvania, the Pennsylvania Hospital, Penn Presbyterian Hospital, and Chester County Hospital. The deliveries were identified using a previously developed algorithm called Method to Acquire Delivery Date Information from Electronic Health Records (MADDIE) [23]. The MADDIE identified deliveries occurred between 2010 and 2017. The outcome of interest was MB as determined by the International Classification of Diseases, 9th Revision, Clinical Modification and International Classification of Diseases (ICD), 10th Revision, billing codes. We used only the MB codes assigned at the time of delivery (ie, we did not include MB if coded during the pregnancy and not at the time of birth). The total list of codes used to define our outcome is provided in Multimedia Appendix 1. MB differs slightly from multifetal pregnancies in that MB indicates that at the time of birth, the pregnancy consisted of multiple fetuses. Therefore, vanishing twin syndrome and other pregnancy conditions or procedures that reduce the number of fetuses before birth were not assessed in this study [24,25]. We obtained a waiver of consent, as this study included retrospective EHR data analysis without further contact with patients.

### Ethics Approval

This study was approved by the institutional review board of the University of Pennsylvania (#828000).

### Adjustment for Known Associations of MB

Although a majority of twin births result from natural conception, the incidence of twins and other higher order multifetal pregnancy resulting from superovulation and ART is 20 times greater than the incidence from natural conception [26]. Therefore, we adjusted for ICD, 9th Revision and ICD, 10th Revision billing codes for ART-resulting pregnancy and infertility diagnoses (Multimedia Appendix 2). As ART and infertility diagnoses would likely be assigned both before pregnancy and during pregnancy, we assigned patients as having ART and infertility if they received any of the corresponding ICD codes between 315 days before delivery and the expected date of delivery.

### Drug Classification (Exposure Classification)

We mapped all inpatient and outpatient medications from Epic and other EHR systems to RxNorm using a previously described method [27]. In short, medications are mapped to the best match to RxNorm, which is limited to the granularity of the ingredient concept. We defined a *preconception/first trimester exposure* as any medication prescription occurring from 275 days before delivery to 215 days before delivery to capture medications slightly before conception and the first trimester of pregnancy. As ART and fertility medications are often prescribed around the time of conception, we chose this window. Most multifetal pregnancies result in preterm birth and are often completed in <270 days after conception. Therefore, we chose the window of 275-215 days before birth to capture the preconception and periconception window where ART and fertility medications are likely to be used.

We manually annotated the complete list of medications, adding the following elements: generic name, medication type, specific medication type, US Federal Drug Agency pregnancy category, associated comorbidities, and associations with pregnancy outcome treatment. We manually annotated this list because many drugs used in fertility treatments are used off-label; therefore, standardized medical terminology systems would be ineffective in capturing those use cases [28]. We referred to the database [29], RxNav, and a reference guide to fetal and neonatal risk [30] to assign medication use categories to each medication as appropriate. The database [29] is sourced from several medication information suppliers, including Wolters Kluwer Health, the American Society of Health System Pharmacists, Cerner Multum, IBM Watson Micromedex, and Mayo Clinic. Medications used for ART and infertility treatment were defined by the Society for Assisted Reproductive Technology (SART) consumer information and practice guidelines [31]. We grouped medications that were generic and brand names into one to evaluate the effect of the primary ingredient on the birth outcome. Next, we limited the medication list to medications prescribed to at least five patients during the defined exposure time.

### Statistical Analysis: MWAS of MB

We constructed 3 logistic regression models with MB as our outcome of interest (binary outcome, 0 or 1), and the effect of each medication on the outcome was assessed separately (each medication exposure was a binary variable, coded 0 or 1). The analysis was performed using the general linear model function in R. The control group for each medication comprised all patients without exposure to the target medication (coded as 0), including patients who had no exposure to medications in the EHR data. Consequently, each target medication had its own control group. We adjusted for 3 known confounders of MB: maternal age (encounter age), ART-resulting pregnancy diagnoses (0 or 1), and infertility diagnoses (0 or 1; Multimedia Appendix 2). A total of three models were constructed: (1) model 1 (no adjustment), (2) model 2 (adjustment for maternal age), and (3) model 3 (adjustment for maternal age, ART-resulting pregnancy, and infertility diagnosis). Diagnoses for ART-resulting pregnancy and infertility were considered in model 3 to account for potential missing prescription data for fertility medical treatment. We reported significant medications (nominal *P*<.05; Bonferroni-adjusted *P*<.05) given the multiple testing that we were performing and calculated odds ratios (ORs) with 95% CIs.

### Validation of MWAS and Determining Novel Medications Associated With MB

Significant medications (*P*<.05) with nominal *P* values and Bonferroni-adjusted *P* values were evaluated on performance to capture medications used in ART and infertility treatment with binary classification. As previously stated, ART use is an established factor in multifetal pregnancy; therefore, these medications are likely to be associated with MB. The analysis was limited to the medications captured within the defined medication exposure window. Using confusion matrices, we calculated precision, sensitivity, specificity, accuracy, and F1 score (Multimedia Appendix 3).

We categorized medications with significant nominal *P* values into three categories: (1) fertility medications used in ART, (2) medications used for comorbidities associated with MB, and (3) medications not associated with MB in the current literature.

## Results

### Cohort Characteristics

**Figure 1:**
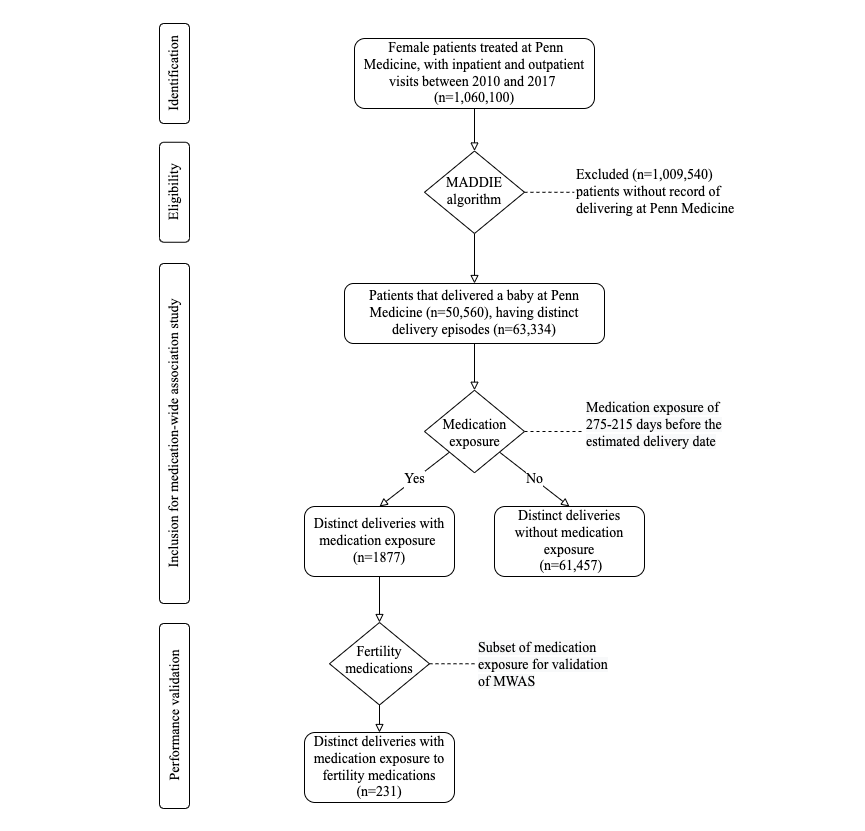
Retrospective cohort selection process. MADDIE: Method to Acquire Delivery Date Information from Electronic Health Records; MWAS: medication-wide association study.

We obtained EHR data from 1,060,100 female patients treated at Penn Medicine, with inpatient and outpatient visits between 2010 and 2017. A previously developed algorithm called MADDIE identified 50,560 patients who delivered a baby at Penn Medicine having 63,334 distinct deliveries [23]. Figure 1 illustrates the study selection process of the cohort. As shown in Figure 1, our cohort contained 63,334 pregnancies delivered between 2010 and 2017 at Penn Medicine, which was determined by the previously developed MADDIE algorithm [23]. We found that 1562 pregnancies included multiples (eg, twins, triplets, and other higher order multiples), amounting to 2.47% (1562/63,334) of our cohort. We found that of 63,334 pregnancies, 1877 (2.96%) had a recorded prescription medication exposure during the defined exposure time. Furthermore, we found that 5.5% (86/1562) MB pregnancies had a recorded prescription medication exposure during pregnancy.

### Drug Classification (Exposure Classification)

We manually annotated 123 medications that were prescribed during the preconception period and the first trimester period of 1877 pregnancies of the 63,334 (2.96%) total distinct deliveries in our cohort (Table 1). These 123 medications belonged to 25 broad drug classes. In our cohort, 15 medications that are typically used as part of fertility treatment were prescribed during pregnancy (Multimedia Appendix 4) [21,22]. Pregnancies with fertility medication exposure (231/63,334, 0.4%) are described in Table 1; the mean age difference (34.6, SD 4.0 years) and the higher incidence of MB (37/231, 16%), ART (16/231, 6.9%), and infertility (4/231, 1.7%) diagnoses are notable, as expected with patients using fertility medication.

Aside from fertility medications, the list contained several types of pain (15/123, 12.2%), antibiotic (11/123, 8.9%), and antihistamine medications (8/123, 6.5%). Most of the extracted medications were not formally assigned (48/123, 39%), followed by category C (31/123, 25.2%) and category B (24/123, 19.5%) medications. As expected, fewer medications were categorized as category A (2/123, 1.6%) and category D (5/123, 4.1%). We found 9.8% (12/123) of medications were categorized as category X, contraindicated in pregnancy, medications—all of which are medications indicated for fertility treatment, contraception, or other indications in obstetrics and gynecology practice.

**Table 1:** Retrospective cohort medication exposure data.

		Total distinct deliveries (N=63,334)	No prescription medication exposure (n=61,457)	Prescription medication exposure^a^ (n=1877)	Fertility medication^b^ exposure (n=231)
**Pregnancy outcome, n (%)**					
	Multiple birth^c^	1562 (2.47)	1476 (2.4)	86 (4.58)	37 (16)
**Diagnosis, n (%)**					
	Assisted reproductive technology^d^	246 (0.39)	218 (0.35)	28 (1.49)	16 (6.9)
	Infertility^e^	48 (0.08)	39 (0.06)	9 (0.48)	4 (1.7)
Maternal age, mean (SD)		29.5 (6.1)	29.5 (6.1)	30.5 (5.7)	34.6 (4.0)

^a^Prescription medication exposure is during the preconception period and the first trimester period only in this cohort.

^b^Multimedia Appendix 4 provides a list of medications with indication for infertility treatment; note that this is a subset of patients with prescription medication exposure.

^c^Multiple birth determined by International Classification of Diseases (ICD) codes shown in Multimedia Appendix 1.

^d^Pregnancy resulting from assisted reproductive was determined by the ICD codes shown in Multimedia Appendix 2.

^e^Infertility diagnosis determined by ICD codes shown in Multimedia Appendix 2.

### MWAS: MB

**Figure 2:**
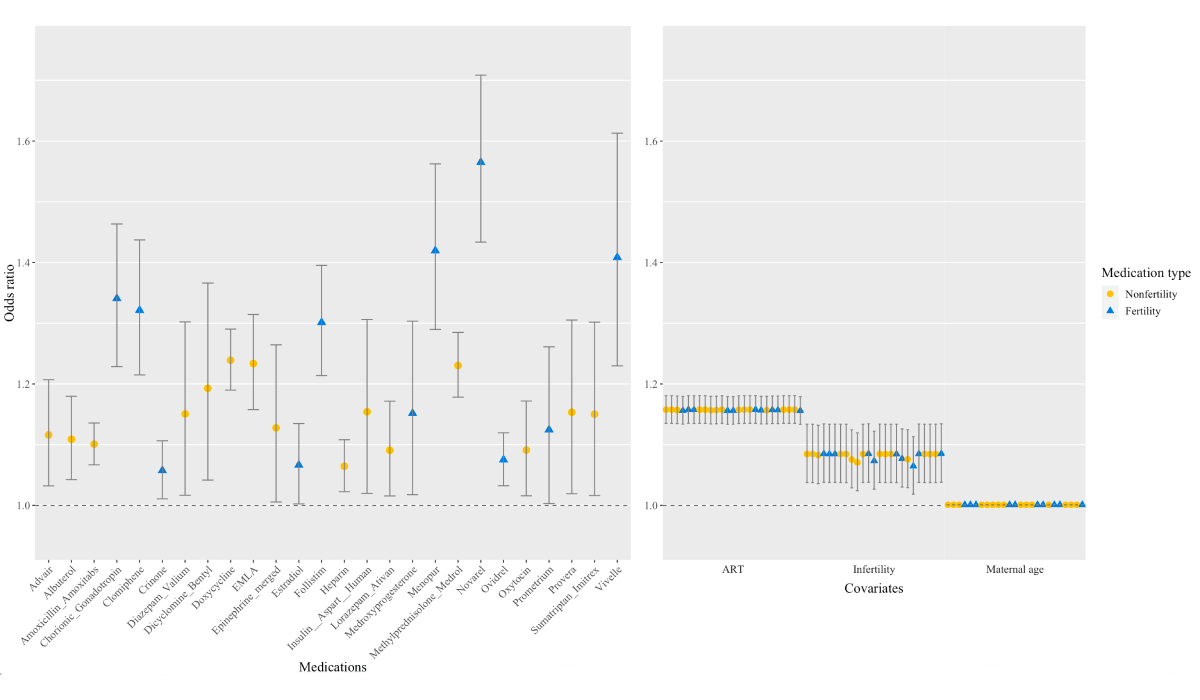
Medications and covariates significantly associated with multiple birth, using odds ratio (95% CIs). Medication names found significant in our logistic regression model 3 (*P*<.05) are categorized by drug classification. Odds ratio and CIs are plotted for the covariates in model 3, by each medication: assisted reproductive technology (ART)–resulting pregnancy, infertility diagnosis, and maternal age. Fertility medications are indicated in blue.

In Figure 2, the significant medications (*P*<.001 to *P*=.04) from our fully adjusted model (ie, model 3) are shown with ORs (95% CIs) in a forest plot. The results for all 3 models are presented in Multimedia Appendix 5. Several fertility treatment medications have higher ORs in comparison, namely, Vivelle, Novarel, Menopur, Follistim, clomiphene, and chorionic gonadotropin. The medication class with the highest number of drugs associated with MB (*P*<.001 to *P*=.04) was fertility treatment (11/123, 8.9%) prescriptions. The forest plot in Figure 2 illustrates the OR (95% CI) of the significant medications by the covariates in model 3, where an association with ART-resulting pregnancy and infertility diagnoses is shown. The resulting ORs with 95% CIs are listed in Multimedia Appendix 6 for significant (*P*<.001 to *P*=.04) and nonsignificant (*P*=.5 to *P*=.98) medications.

### Validation Set Performance

Validation set performance was evaluated for our fully adjusted model (model 3). Of the 123 medications extracted, we found 26 (21.1%) medications nominally associated with MB (*P*<.001 to *P*=.04) and 11 (8.9%) medications associated with MB using the Bonferroni adjustment (*P*<.001 to *P*=.04). Multimedia Appendix 5 provides the confusion tables from the performance analysis of all 3 models. Using the Bonferroni correction method, 57% (8/14) fertility medications were captured, whereas 79% (11/14) were captured using the raw or nominal *P* value (Multimedia Appendix 7); therefore, sensitivity performance was greater using noncorrected *P* values (Table 2). This indicates the utility of using nominal *P* values in exploratory MWAS.

**Table 2:** Performance validation of assisted reproductive technology medications in medication-wide association study.

		Performance metric^a^				
		Sensitivity	Specificity	Accuracy	Precision	F1 score
**Model 1: no adjustment**						
	*P* value	.80	.84	.84	.41	.54
	*P* value with Bonferroni adjustment	.47	.96	.90	.64	.54
**Model 2: adjustment for maternal age^b^**						
	*P* value	.73	.85	.84	.41	.53
	*P* value with Bonferroni adjustment	.47	.96	.90	.64	.50
**Model 3: adjustment for maternal age and assisted reproductive technology diagnosis^c^ and infertility diagnosis^d^**						
	*P* value	.73	.86	.85	.42	.53
	*P* value with Bonferroni adjustment	.40	.96	.89	.60	.48

^a^Performance metrics were calculated using formulas shown in Multimedia Appendix 3.

^b^Maternal age determined by age at delivery encounter.

^c^Pregnancy resulting from assisted reproductive technology determined by the International Classification of Diseases codes shown in Multimedia Appendix 2.

^d^Infertility diagnosis determined by the International Classification of Diseases codes shown in Multimedia Appendix 2.

### Known, Confounding, and Unknown Associations

**Figure 3:**
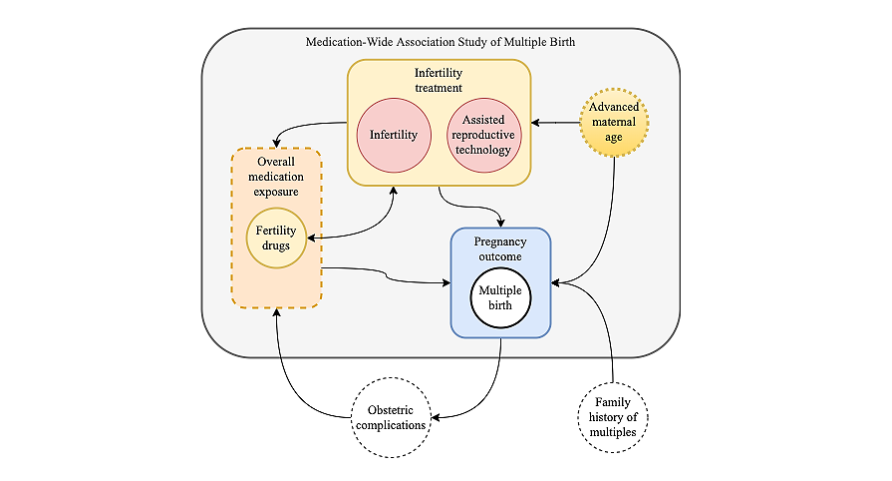
Conceptual schema for medication-wide association study (MWAS) analyses on multiple birth. Confounding relationships for medication-outcome associations are illustrated. Within the MWAS, we adjust for maternal age, infertility diagnosis, and assisted reproductive technology–resulting pregnancy diagnosis. The study does not adjust for all known associations of multiple birth such as obstetric complications or family history of multiples. The validation of the MWAS models observed performance in capturing fertility medication exposure.

Prescription medications associated with comorbidities of fertility and ART treatment were found, as well as medications that may be used for obstetric complications related to multifetal pregnancy care. Of the 26 significant medications using nominal *P* value, 11 (42%) were potential fertility treatment medications; 12 (46%) were associated with infertility and ART use or complications associated with multifetal pregnancy; and 3 (12%) were not previously associated with MB, ART, or fertility-related problems (ie, novel findings or unexpected agents; Table 3). As shown in Figure 3, the validation set included medications used for infertility treatment (medications listed in Multimedia Appendix 4). Nevertheless, the MWAS for MB included confounding medication exposure during multifetal pregnancy, prescribed for (1) treatment of comorbidities of infertility and ART use and (2) treatment of obstetric complications of multifetal pregnancy. Medications associated with ART treatment were associated with MB even after adjusting for ART procedure codes and infertility diagnosis codes. Although 3 asthma medications were found to be significant, previous studies showed mixed results when examining the relationship between asthma, asthma medication use, and fertility [32-34]. The association between irritable bowel disease (IBD) and fertility is complex; patients with quiescent IBD have fertility rates comparable with those of the general population [28], whereas patients with an active disease or those who had undergone a pelvic surgery may have reduced fertility [30]. Overall, we found 3 medications not previously reported to be associated with an increased risk of MB following prescription during the preconception and periconceptional period: sumatriptan and imitrex (*P*=.03), oxytocin (*P*=.02), and lorazepam and ativan (*P*=.02).

**Table 3:** Medications associated with multiple birth after adjustment for assisted reproductive technology, infertility, and maternal age (model 3).

Indicated comorbidity			Generic medication name or names		Medications associated with multiple birth, n (%)
**Associated with infertility and assisted reproductive technology**					
	Assisted reproductive technology treatment	EMLA, methylprednisolone, diazepam, amoxicillin, doxycycline, and medroxyprogesterone acetate		6 (23)	
	Asthma	Albuterol, fluticasone propionate and salmeterol, and epinephrine		3 (12)	
	Irritable bowel disease	Dicyclomine		1 (4)	
**Associated with multifetal pregnancy**					
	Cardiovascular-related diagnoses (gestational hypertension and thrombosis)	Heparin		1 (4)	
	Gestational diabetes mellitus	Insulin aspart, human		1 (4)	
Not previously associated with multiple birth, assisted reproductive technology, or fertility-related problems			Sumatriptan, oxytocin, and lorazepam		3 (12)

## Discussion

### Overview

We applied 3 logistic regression models to retrospective EHR data of a cohort of patients who delivered at Penn Medicine between 2010 and 2017 (n=63,334) to explore potential associations between the medications prescribed during the preconception and first trimester period (binary variable) and the occurrence of MB (binary outcome). We discuss the results of our MWAS from our fully adjusted model that was adjusted for age and ART and infertility diagnosis (model 3) on MB for all associations revealed using nominal.

### Reason for Conducting an MWAS on MB

The application of an MWAS approach to MB allows the analysis of medications used outside the scope of obstetric treatment, capturing comorbidities that may increase the risk of the outcome. Not all of this is known, as MB is more commonly used as an adjustment for analysis of other pregnancy outcomes of interest. Off-label use is common in pregnancy and infertility treatment [28,35]. Our MWAS approach with annotation of known off-label uses can further improve the identification of comorbidities associated with MB (eg, infertility and subsequent ART use). Research into the side effects of medications is more focused on adverse outcomes than MB, notwithstanding the risks of multifetal pregnancy.

The graph in Multimedia Appendix 8 illustrates the overlap between patients with the respective fertility diagnoses and fertility medication prescriptions in our cohort. This also demonstrates that many patients with fertility or ART medications were not assigned the corresponding diagnostic code, indicating that fertility studies using EHR data should include medication history to fully capture affected patients. A recently enhanced algorithm to detect ectopic pregnancy in the EHR used diagnosis and procedure codes as well as medication exposure [36]. The complete picture of a patient’s medical encounters during pregnancy is likely not captured in the EHR of a health care system (eg, engagement with more than one health care system, over-the-counter medications, etc). Adjustment for fertility diagnoses and pregnancies resulting from ART treatments may not truly represent patients undergoing ART and fertility treatment if the diagnosis codes are used without the inclusion of medication histories. The same is true when only fertility medications are observed, especially medications with multiple indications for care in obstetrics and gynecology. Fertility treatment, meaning without eggs or embryos handled, may involve medical treatment (eg, clomiphene and gonadotropins) that increases the chances of multifetal pregnancy. Pregnancies resulting from fertility treatment do not necessarily indicate ART use; therefore, EHR may not reflect ART use diagnosis. The EHR should include an infertility diagnosis in these cases, but we found that in many instances, both infertility diagnoses and ART use codes were absent from those receiving these medications (Multimedia Appendix 8). Using diagnosis codes and medication exposure should allow for better capture of MB in comparison with using only one or the other.

### EHR for MWAS Versus SART Database (or Other ART Cohort Database)

MB outcomes can also be observed in ART cohort databases, such as the SART Clinical Outcomes Reporting System. However, fertility medication treatment without the intention of egg retrieval will not necessarily be captured within such databases, as they are beyond the scope of ART. Moreover, not all multifetal pregnancies result from infertility treatment and ART. Finally, such databases are reported from ART clinics and are not necessarily representative of all the medications prescribed during pregnancy. An ART cohort database may have a wealth of data elements specific to ART treatment; however, these data are reported using inconsistent methods, often from a variety of reporting services [37]. In contrast, EHRs may also have missing prescription information due to offsite care; however, the scope of captured health information is likely more comprehensive overall than that of an ART clinic because it includes medications for comorbidities and other aspects of patients’ care that may be overlooked by ART specialists.

### Medication Exposure During the Preconception and First Trimester Period

We found that 5.51% (86/1562) MB pregnancies had prescription medication exposure during pregnancy. Therefore, pregnancies resulting in MB were more likely to have recorded prescription medication during the preconception and first trimester period. This is consistent with (1) the fact that ART often uses medications early on to induce pregnancy [20] and (2) multifetal pregnancies are at higher risk of pregnancy complications [21] and therefore may be more likely to receive prescription medication treatment. Moreover, a higher proportion of MB (37/231, 16%) was found for those exposed to fertility medications in comparison with the occurrence of MB (1562/63,334, 2.47%) for the overall cohort.

### Our Evaluation Using Known Fertility and ART Medications That Increase the Risk of MB Is Not Perfect

#### Overview

To assess the ability of our MWAS to capture medications that increase the risk of MB, we used medications that are known to increase an individual’s chance of conceiving and have been implicated in increasing the risk of MB in the literature (Multimedia Appendix 4). We know that this list of medications is incomplete (and hence part of the reason for this study), but we wanted to understand how many known medications we were able to capture using our MWAS approach. The indication of medication prescribed is not necessarily straightforward; without observing clinical notes and ICD codes from an encounter, there are often several therapeutic uses for which the medication could have been prescribed (eg, progesterone). More context and research are required to understand the discovered associations further. Although known fertility medications were missed by our approach (3/14, 21%), we observed a large number of drugs not known to be associated with MB with insignificant nominal *P* values (93/123, 75.6%), which is comforting. We observed drugs used in fertility treatment (11/26, 42%) and drugs known to be associated with multifetal pregnancy (12/26, 46%), along with 3 (11%) novel associations. Associated comorbidities of infertility overlap with obstetric complications during multifetal pregnancy, including diabetes mellitus, cardiovascular disease, thyroid dysregulation, and liver dysregulation.

#### Medications Associated With Infertility and ART

Medications used in fertility treatment themselves may be captured solely because of reverse causation, although they do not have a truly strong association with multifetal pregnancy. Several medications may be prescribed during IVF treatment cycles for preventive care or other indications, including antibiotics (doxycycline and amoxicillin), a corticosteroid (methylprednisolone), pain management (EMLA), progestin-induced menstruation (medroxyprogesterone acetate), and conscious sedation (diazepam) [38,39].

ART and ovulation induction procedures are used for fertility treatment. A comprehensive review of infertility comorbidities in women suggests that infertility is a complex health care issue, and women with infertility are at a higher risk of psychiatric disorders and endometrial cancer [40]. Infertility and fertility treatment are associated with other pathologies, such as polycystic ovarian syndrome, endometriosis, thyroid disorders, breast cancer, cardiovascular disease, metabolic syndrome, diabetes mellitus, and liver dysfunction [41].

Medications associated with comorbidities of infertility were identified, including treatments for asthma and IBD. Research shows that women with asthma have higher pregnancy losses [32] and a prolonged time to pregnancy [33]; in contrast, some studies have shown no association [34]. Bronchodilators (albuterol, epinephrine, and fluticasone propionate or salmeterol) may be pharmacological treatments for asthma, which has been linked to a prolonged time to pregnancy and is associated with a higher need for fertility treatment among women aged ≥35 years [42]. In addition, a retrospective study on asthma during pregnancy in Sweden found that women hospitalized for asthma had a higher risk of twinning [43]. Dicyclomine is used to treat IBD; however, the association between IBD and fertility is complex, and patients with quiescent IBD have fertility rates comparable with those of the general population [44], whereas patients with an active disease or those who had undergone a pelvic surgery may have reduced fertility [45].

#### Medications Associated With Obstetric Complications During Multifetal Pregnancy

Medications identified by the MWAS may be prescribed for obstetric complications associated with multifetal pregnancy. These pregnancies are at an increased risk of obstetric complications, such as preterm birth, placental problems, gestational diabetes mellitus, anemia, and preeclampsia. Owing to the time exposure range, medications typically used to treat complications typically past the first trimester of pregnancy were not captured by the MWAS. One antidiabetic medication (ie, insulin aspart, human) was identified; however, other forms of insulin and the insulin sensitizer metformin were not identified as significant. A single antithrombotic medication, heparin, was identified, but other anticoagulants and cardiovascular-related medications were not identified in our models.

### Novel Findings of Medications Associated With MB

Migraines have a high incidence in obstetrics; one migraine pharmacological treatment (sumatriptan) was found to be associated with MB. An association between migraine history and development of ovarian hyperstimulation syndrome may indicate the risk of multifetal pregnancy [46], as ovarian hyperstimulation syndrome–complicated pregnancy is linked to a higher incidence of MB [47]. However, further research is required to understand the biological mechanisms, if any, underlying this association. Oxytocin could be associated with MB because of prior pregnancy delivery episodes (as oxytocin is used during labor), indicating a short time between pregnancies.

### Limitations and Future Work

Although the World Health Organization’s anatomical therapeutic chemical classification system is applicable, the proportion of RxNorm drugs mapped to anatomical therapeutic chemicals would result in fewer medications being included in the analysis. However, the therapeutic use of the medications has not been explicitly determined. Medications classified as fertility related are based on the SART references; however, without discrete indications, they potentially underpower performance in the validation process. In addition, a major limitation of using standard pharmacology and drug-related terminologies is that approximately 11% of medications used in women’s health are off-label [48]. This includes several popular medications commonly used in the obstetrics and gynecology domain [49-51]. Use of off-label medications requires manual review of medications, which is laborious. Manual review and classification of the prescription medications were conducted by an informaticist (LD) and not a pharmacologist. Several extracted prescriptions were available over the counter (38/123, 30.9%); therefore, exposure to such drugs is likely underrepresented in our cohort. Potentially highly related variables were not considered in the analysis, introducing a possible omitted variable bias (eg, drug dose, drug form, route of application, and temporal component of exposure). Medication exposure of 275-215 days before subsequent delivery may likely include medication exposure before conception (ie, prior pregnancy delivery episodes), especially regarding the length of gestation due to preterm birth. We did not ensure that medication exposure occurred before conception; therefore, the medications associated with multifetal pregnancies in this study are not causal in nature. Unexpected agents and probable confounding medications require further adjustment in the MWAS technique to provide more reliable, meaningful results.

### Conclusions

Our research demonstrates opportunities in using an MWAS approach with EHR data to explore agents previously unknown to be associated with MB outcomes. The results indicated that a number of medications used in ART and infertility treatment were associated with an increased incidence of MB likely due to multifetal pregnancy, as expected. Using these medications as our gold standard, we found that our algorithm had an accuracy of 85% and 89%, using nominal *P* values and Bonferroni-adjusted *P* values, respectively. Sensitivity and F1 score were improved using nominal *P* values in comparison with Bonferroni-adjusted *P* values, indicating the applicability of nominal *P* values in exploratory MWAS studies. A total of 6 novel agents were linked to MB, with the remaining 20 medications potentially linked to the comorbidities of infertility, ART use, and obstetric complications during multifetal pregnancy. The MWAS approach can facilitate hypothesis-driven data exploration, informing the adjustments needed in the models in further research. Our approach also highlights the importance of exploring medication histories, as many patients receiving ART and fertility treatments do not have corresponding diagnosis codes indicating treatment. If medication information was not used, these patients were mistakenly labeled as having not received ART and infertility treatment. This underscores the importance of multidata modalities in retrospective EHR studies, especially for those exploring the effects and outcomes related to pregnancy.

## Appendix

Multimedia Appendix 1 International Classification of Diseases (ICD)-9 and ICD-10 codes to capture multiple birth.

Multimedia Appendix 2 International Classification of Diseases (ICD)-9 and ICD-10 codes used to identify comorbidity diagnosis from the electronic health record for model adjustment.

Multimedia Appendix 3 Formulas for validation.

Multimedia Appendix 4 Medications that can be indicated for infertility treatment.

Multimedia Appendix 5 Medication-wide association study results for models 1, 2, and 3.

Multimedia Appendix 6 Odds ratio with 95% CIs for observed prescription of medications.

Multimedia Appendix 7 Performance analysis confusion tables.

Multimedia Appendix 8 Graph of patients with assisted reproductive technology or fertility diagnosis and patients with fertility medication prescriptions.

## Abbreviations

ART: assisted reproductive technology
EHR: electronic health record
IBD: irritable bowel disease
ICD: International Classification of Diseases
IVF: in vitro fertilization
MADDIE: Method to Acquire Delivery Date Information from Electronic Health Records
MB: multiple birth
MWAS: medication-wide association study
OR: odds ratio
SART: Society for Assisted Reproductive Technology
