# Supplementary material for: Medication-Wide Association Study Using Electronic Health Record Data of Prescription Medication Exposure and Multifetal Pregnancies: Retrospective Study
Source: JMIR Med Inform. 2022 Jun 7;10(6):e32229. doi: 10.2196/32229 (PMC9214620; doi:10.2196/32229)
Supplement: Multimedia Appendix 1 [file medinform_v10i6e32229_app1.docx]

**Appendix 1**. ICD 9 & ICD 10 Codes to Capture Multiple Birth

| Code | Version | Code Description |
| --- | --- | --- |
| 651.01 | ICD-9 | Twin pregnancy, delivered, with or without mention of antepartum condition |
| 651.11 | ICD-9 | Triplet pregnancy, delivered, with or without mention of antepartum condition |
| 651.21 | ICD-9 | Quadruplet pregnancy, delivered, with or without mention of antepartum condition |
| 651.31 | ICD-9 | Twin pregnancy with fetal loss and retention of one fetus, delivered, with or without mention of antepartum condition |
| 651.41 | ICD-9 | Triplet pregnancy with fetal loss and retention of one or more fetus(es), delivered, with or without mention of antepartum condition |
| 651.51 | ICD-9 | Quadruplet pregnancy with fetal loss and retention of one or more fetus(es), delivered, with or without mention of antepartum condition |
| 651.61 | ICD-9 | Other multiple pregnancy with fetal loss and retention of one or more fetus(es), delivered, with or without mention of antepartum condition |
| 651.71 | ICD-9 | Multiple gestation following (elective) fetal reduction, delivered, with or without mention of antepartum condition |
| 651.81 | ICD-9 | Other specified multiple gestation, delivered, with or without mention of antepartum condition |
| 651.91 | ICD-9 | Unspecified multiple gestation, delivered, with or without mention of antepartum condition |
| 652.61 | ICD-9 | Multiple gestation with malpresentation of one fetus or more, delivered, with or without mention of antepartum condition |
| 660.51 | ICD-9 | Locked twins, delivered, with or without mention of antepartum condition |
| 662.31 | ICD-9 | Delayed delivery of second twin, triplet, etc., delivered, with or without mention of antepartum condition |
| 678.11 | ICD-9 | Fetal conjoined twins, delivered, with or without mention of antepartum condition |
| V27.2 | ICD-9 | Outcome of delivery, twins, both liveborn |
| V27.3 | ICD-9 | Outcome of delivery, twins, one liveborn and one stillborn |
| V27.4 | ICD-9 | Outcome of delivery, twins, both stillborn |
| V27.5 | ICD-9 | Outcome of delivery, other multiple birth, all liveborn |
| V27.6 | ICD-9 | Outcome of delivery, other multiple birth, some liveborn |
| V27.7 | ICD-9 | Outcome of delivery, other multiple birth, all stillborn |
| V31.00 | ICD-9 | Twin birth, mate liveborn, born in hospital, delivered without mention of cesarean section |
| V31.01 | ICD-9 | Twin birth, mate liveborn, born in hospital, delivered by cesarean section |
| V31.1 | ICD-9 | Twin birth, mate liveborn, born before admission to hospital |
| V31.2 | ICD-9 | Twin birth, mate liveborn, born outside hospital and not hospitalized |
| V32.00 | ICD-9 | Twin birth, mate stillborn, born in hospital, delivered without mention of cesarean section |
| V32.01 | ICD-9 | Twin birth, mate stillborn, born in hospital, delivered by cesarean section |
| V32.1 | ICD-9 | Twin birth, mate stillborn, born before admission to hospital |
| V32.2 | ICD-9 | Twin birth, mate stillborn, born outside hospital and not hospitalized |
| V33.00 | ICD-9 | Twin birth, unspecified whether mate liveborn or stillborn, born in hospital, delivered without mention of cesarean section |
| V33.01 | ICD-9 | Twin birth, unspecified whether mate liveborn or stillborn, born in hospital, delivered by cesarean section |
| V33.1 | ICD-9 | Twin birth, unspecified whether mate liveborn or stillborn, born before admission to hospital |
| V33.2 | ICD-9 | Twin birth, unspecified whether mate liveborn or stillborn, born outside hospital and not hospitalized |
| V34.00 | ICD-9 | Other multiple birth (three or more), mates all liveborn, born in hospital, delivered without mention of cesarean section |
| V34.01 | ICD-9 | Other multiple birth (three or more), mates all liveborn, born in hospital, delivered by cesarean section |
| V34.1 | ICD-9 | Other multiple birth (three or more), mates all liveborn, born before admission to hospital |
| V34.2 | ICD-9 | Other multiple birth (three or more), mates all liveborn, born outside hospital and not hospitalized |
| V35.00 | ICD-9 | Other multiple birth (three or more), mates all still born, born in hospital, delivered without mention of cesarean section |
| V35.01 | ICD-9 | Other multiple birth (three or more), mates all still born, born in hospital, delivered by cesarean section |
| V35.1 | ICD-9 | Other multiple birth (three or more), mates all stillborn, born before admission to hospital |
| V35.2 | ICD-9 | Other multiple birth (three or more), mates all stillborn, born outside of hospital and not hospitalized |
| V36.00 | ICD-9 | Other multiple birth (three or more), mates liveborn and stillborn, born in hospital, delivered without mention of cesarean section |
| V36.01 | ICD-9 | Other multiple birth (three or more), mates liveborn and stillborn, born in hospital, delivered by cesarean section |
| V36.1 | ICD-9 | Other multiple birth (three or more), mates liveborn and stillborn, born before admission to hospital |
| V36.2 | ICD-9 | Other multiple birth (three or more), mates liveborn and stillborn, born outside hospital and not hospitalized |
| V37.00 | ICD-9 | Other multiple birth (three or more), unspecified whether mates liveborn or stillborn, born in hospital, delivered without mention of cesarean section |
| V37.01 | ICD-9 | Other multiple birth (three or more), unspecified whether mates liveborn or stillborn, born in hospital, delivered by cesarean section |
| V37.1 | ICD-9 | Other multiple birth (three or more), unspecified whether mates liveborn or stillborn, born before admission to hospital |
| V37.2 | ICD-9 | Other multiple birth (three or more), unspecified whether mates liveborn or stillborn, born outside of hospital |
| O30.02 | ICD-10 | Conjoined twin pregnancy |
| O30.021 | ICD-10 | Conjoined twin pregnancy, first trimester |
| O30.022 | ICD-10 | Conjoined twin pregnancy, second trimester |
| O30.023 | ICD-10 | Conjoined twin pregnancy, third trimester |
| O30.029 | ICD-10 | Conjoined twin pregnancy, unspecified trimester |
| O60.10X2 | ICD-10 | Preterm labor with preterm delivery, unspecified trimester, fetus 2 |
| O60.10X3 | ICD-10 | Preterm labor with preterm delivery, unspecified trimester, fetus 3 |
| O60.10X4 | ICD-10 | Preterm labor with preterm delivery, unspecified trimester, fetus 4 |
| O60.10X5 | ICD-10 | Preterm labor with preterm delivery, unspecified trimester, fetus 5 |
| O60.10X9 | ICD-10 | Preterm labor with preterm delivery, unspecified trimester, other fetus |
| O60.12X2 | ICD-10 | Preterm labor second trimester with preterm delivery second trimester, fetus 2 |
| O60.12X3 | ICD-10 | Preterm labor second trimester with preterm delivery second trimester, fetus 3 |
| O60.12X4 | ICD-10 | Preterm labor second trimester with preterm delivery second trimester, fetus 4 |
| O60.12X5 | ICD-10 | Preterm labor second trimester with preterm delivery second trimester, fetus 5 |
| O60.12X9 | ICD-10 | Preterm labor second trimester with preterm delivery second trimester, other fetus |
| O60.13X2 | ICD-10 | Preterm labor second trimester with preterm delivery third trimester, fetus 2 |
| O60.13X3 | ICD-10 | Preterm labor second trimester with preterm delivery third trimester, fetus 3 |
| O60.13X4 | ICD-10 | Preterm labor second trimester with preterm delivery third trimester, fetus 4 |
| O60.13X5 | ICD-10 | Preterm labor second trimester with preterm delivery third trimester, fetus 5 |
| O60.13X9 | ICD-10 | Preterm labor second trimester with preterm delivery third trimester, other fetus |
| O60.14X2 | ICD-10 | Preterm labor third trimester with preterm delivery third trimester, fetus 2 |
| O60.14X3 | ICD-10 | Preterm labor third trimester with preterm delivery third trimester, fetus 3 |
| O60.14X4 | ICD-10 | Preterm labor third trimester with preterm delivery third trimester, fetus 4 |
| O60.14X5 | ICD-10 | Preterm labor third trimester with preterm delivery third trimester, fetus 5 |
| O60.14X9 | ICD-10 | Preterm labor third trimester with preterm delivery third trimester, other fetus |
| O60.20X2 | ICD-10 | Term delivery with preterm labor, unspecified trimester, fetus 2 |
| O60.20X3 | ICD-10 | Term delivery with preterm labor, unspecified trimester, fetus 3 |
| O60.20X4 | ICD-10 | Term delivery with preterm labor, unspecified trimester, fetus 4 |
| O60.20X5 | ICD-10 | Term delivery with preterm labor, unspecified trimester, fetus 5 |
| O60.20X9 | ICD-10 | Term delivery with preterm labor, unspecified trimester, other fetus |
| O60.22X2 | ICD-10 | Term delivery with preterm labor, second trimester, fetus 2 |
| O60.22X3 | ICD-10 | Term delivery with preterm labor, second trimester, fetus 3 |
| O60.22X4 | ICD-10 | Term delivery with preterm labor, second trimester, fetus 4 |
| O60.22X5 | ICD-10 | Term delivery with preterm labor, second trimester, fetus 5 |
| O60.22X9 | ICD-10 | Term delivery with preterm labor, second trimester, other fetus |
| O60.23X2 | ICD-10 | Term delivery with preterm labor, third trimester, fetus 2 |
| O60.23X3 | ICD-10 | Term delivery with preterm labor, third trimester, fetus 3 |
| O60.23X4 | ICD-10 | Term delivery with preterm labor, third trimester, fetus 4 |
| O60.23X5 | ICD-10 | Term delivery with preterm labor, third trimester, fetus 5 |
| O60.23X9 | ICD-10 | Term delivery with preterm labor, third trimester, other fetus |
| Z37.2 | ICD-10 | Twins, both liveborn |
| Z37.3 | ICD-10 | Twins, one liveborn and one stillborn |
| Z37.4 | ICD-10 | Twins, both stillborn |
| Z37.5 | ICD-10 | Other multiple births, all liveborn |
| Z37.50 | ICD-10 | Multiple births, unspecified, all liveborn |
| Z37.51 | ICD-10 | Triplets, all liveborn |
| Z37.52 | ICD-10 | Quadruplets, all liveborn |
| Z37.53 | ICD-10 | Quintuplets, all liveborn |
| Z37.54 | ICD-10 | Sextuplets, all liveborn |
| Z37.59 | ICD-10 | Other multiple births, all liveborn |
| Z37.6 | ICD-10 | Other multiple births, some liveborn |
| Z37.60 | ICD-10 | Multiple births, unspecified, some liveborn |
| Z37.61 | ICD-10 | Triplets, some liveborn |
| Z37.62 | ICD-10 | Quadruplets, some liveborn |
| Z37.63 | ICD-10 | Quintuplets, some liveborn |
| Z37.64 | ICD-10 | Sextuplets, some liveborn |
| Z37.69 | ICD-10 | Other multiple births, some liveborn |
| Z37.7 | ICD-10 | Other multiple births, all stillborn |
| Z38.3 | ICD-10 | Twin liveborn infant, born in hospital |
| Z38.30 | ICD-10 | Twin liveborn infant, delivered vaginally |
| Z38.31 | ICD-10 | Twin liveborn infant, delivered by cesarean |
| Z38.6 | ICD-10 | Other multiple liveborn infant, born in hospital |
| Z38.61 | ICD-10 | Triplet liveborn infant, delivered vaginally |
| Z38.62 | ICD-10 | Triplet liveborn infant, delivered by cesarean |
| Z38.63 | ICD-10 | Quadruplet liveborn infant, delivered vaginally |
| Z38.64 | ICD-10 | Quadruplet liveborn infant, delivered by cesarean |
| Z38.65 | ICD-10 | Quintuplet liveborn infant, delivered vaginally |
| Z38.66 | ICD-10 | Quintuplet liveborn infant, delivered by cesarean |
| Z38.68 | ICD-10 | Other multiple liveborn infant, delivered vaginally |
| Z38.69 | ICD-10 | Other multiple liveborn infant, delivered by cesarean |
