# Supplementary material for: Medication-Wide Association Study Using Electronic Health Record Data of Prescription Medication Exposure and Multifetal Pregnancies: Retrospective Study
Source: JMIR Med Inform. 2022 Jun 7;10(6):e32229. doi: 10.2196/32229 (PMC9214620; doi:10.2196/32229)
Supplement: Multimedia Appendix 2 [file medinform_v10i6e32229_app2.docx]

**Appendix 2.** ICD-9 and ICD-10 codes used to identify comorbidity diagnosis from the EHR for model adjustment.

| Group | Code | Version | Code Description |
| --- | --- | --- | --- |
| Infertility | 628 | ICD-9 | Female infertility |
|  | 628.0 | ICD-9 | Infertility, female, associated with anovulation |
|  | 628.1 | ICD-9 | Infertility, female, of pituitary-hypothalamic origin |
|  | 628.2 | ICD-9 | Infertility, female, of tubal origin |
|  | 628.3 | ICD-9 | Infertility, female, of uterine origin |
|  | 628.4 | ICD-9 | Infertility, female, of cervical or vaginal origin |
|  | 628.8 | ICD-9 | Infertility, female, of other specified origin |
|  | 628.9 | ICD-10 | Infertility, female, of unspecified origin |
|  | N97.0 | ICD-10 | Female infertility associated with anovulation |
|  | N97.1 | ICD-10 | Female infertility of tubal origin |
|  | N97.2 | ICD-10 | Female infertility of uterine origin |
|  | N97.8 | ICD-10 | Female infertility of other origin |
|  | N97.9 | ICD-10 | Female infertility, unspecified |
| Pregnancy Resulting from Assisted Reproductive Technology | N98.0 | ICD-10 | Infection associated with artificial insemination |
|  | N98.1 | ICD-10 | Hyperstimulation of ovaries |
|  | O09.811 | ICD-10 | Supervision of pregnancy resulting from assisted reproductive technology, first trimester |
|  | O09.812 | ICD-10 | Supervision of pregnancy resulting from assisted reproductive technology, second trimester |
|  | O09.813 | ICD-10 | Supervision of pregnancy resulting from assisted reproductive technology, third trimester |
|  | O09.819 | ICD-10 | Supervision of pregnancy resulting from assisted reproductive technology, unspecified trimester |
|  | V23.85 | ICD-9 | Pregnancy resulting from assisted reproductive technology |
|  | V26.1 | ICD-9 | Artificial insemination |
|  | V26.42 | ICD-9 | Encounter for fertility preservation counseling |
|  | V26.82 | ICD-9 | Encounter for fertility preservation procedure |
|  | Z31.0 | ICD-10 | Encounter for reversal of previous sterilization |
|  | Z31.41 | ICD-10 | Encounter for fertility testing |
|  | Z31.42 | ICD-10 | Aftercare following sterilization reversal |
|  | Z31.62 | ICD-10 | Encounter for fertility preservation counseling |
|  | Z31.83 | ICD-10 | Encounter for assisted reproductive fertility procedure cycle |
|  | Z31.84 | ICD-10 | Encounter for fertility preservation procedure |
