# Supplementary material for: Medication-Wide Association Study Using Electronic Health Record Data of Prescription Medication Exposure and Multifetal Pregnancies: Retrospective Study
Source: JMIR Med Inform. 2022 Jun 7;10(6):e32229. doi: 10.2196/32229 (PMC9214620; doi:10.2196/32229)
Supplement: Multimedia Appendix 3 [file medinform_v10i6e32229_app3.pdf]

Appendix 3. Formulas for Validation

$$precision = \frac{True\ Positives}{True\ Positives + False\ Positives}$$

$$sensitivity = \frac{True\ Positives}{True\ Positives + False\ Negatives}$$

$$accuracy = \frac{True\ Positives + True\ Negatives}{TP + TN + FP + FN}$$

$$F_1 score = 2 \cdot \frac{precision \cdot sensitivity}{precision + sensitivity}$$

$$specificity = \frac{True\ Negatives}{True\ Negatives + False\ Positives}$$
