# Supplementary material for: Medication-Wide Association Study Using Electronic Health Record Data of Prescription Medication Exposure and Multifetal Pregnancies: Retrospective Study
Source: JMIR Med Inform. 2022 Jun 7;10(6):e32229. doi: 10.2196/32229 (PMC9214620; doi:10.2196/32229)
Supplement: Multimedia Appendix 4 [file medinform_v10i6e32229_app4.docx]

**Appendix 4.** Medications that can be indicated for infertility treatment.

| **Medication Name** | **Generic Name** | **Pregnancy Category^a^** | **Drug Type** | **Treatment Usage^b^** |
| --- | --- | --- | --- | --- |
| Follistim | follitropin | X | Gonadotropin | Induce ovulation |
| Novarel | chorionic gonadotropin (Human) | X |  |  |
| Ovidrel | chorionic gonadotropin (recombinant) | X |  |  |
| Chorionic_Gonadotropin | chorionic gonadotropin | X |  |  |
| Menopur | follicle stimulating hormone / luteinizing hormone | X |  |  |
| progesterone  Prometrium | progesterone | B | Progesterone | Luteal phase support, previous miscarriage |
| Medroxyprogesterone | medroxyprogesterone | X | Progestin | Oral contraceptive prior to the ART^c^ cycle |
| Clomiphene | clomiphene | X | Selective estrogen receptor modulator | Induce ovulation |
| Cabergoline | cabergoline | B | Dopamine receptor agonist | Hyperprolactinemia |
| Vivelle  Estrace  Estradiol | estradiol | X | Estrogen | Menopause symptoms, maturation of endometrial lining |
| Letrozole | letrozole | N | Aromatase inhibitor | Post-menopausal breast cancer (on-label), infertility for PCOS (off-label) |

^a^ US FDA five letter risk categories (since 2015 replaced with PLLR labeling) to indicate the potential of a drug to cause birth defects if used during pregnancy: A (adequate evidence to support safety), B (no adequate studies in humans and animal reproduction studies have failed to demonstrate risk), C (animal reproduction studies show adverse effect, and no adequate studies in humans), D (positive evidence of adverse risk on the fetus in humans), X (contraindicated, studies in animals or humans well demonstrate birth defects risk), N (not formally assigned a category)

^b^ Treatment usage reflects treatment indication in assisted reproductive technology. This includes off-label use of drugs approved for other indications. Accordingly, these medications may be used for other indications. [35]

^c^ART: Assisted Reproductive Technology
