# Supplementary material for: Medication-Wide Association Study Using Electronic Health Record Data of Prescription Medication Exposure and Multifetal Pregnancies: Retrospective Study
Source: JMIR Med Inform. 2022 Jun 7;10(6):e32229. doi: 10.2196/32229 (PMC9214620; doi:10.2196/32229)
Supplement: Multimedia Appendix 6 [file medinform_v10i6e32229_app6.docx]

**Appendix 6.** **Odds ratio with 95% CIs for observed prescription medications.**

| Significance and Medication Class | Generic medication name | Value, n | Pregnancy category^a^ | Odds ratio (95% CI) |
| --- | --- | --- | --- | --- |
| **Significant (*P*<.05)** | | | | |
| **Antibiotic** |  |  |  |  |
|  | Amoxicillin^b^ | 94 | B | 1.10 (1.07-1.14) |
|  | Doxycycline^b^ | 56 | D | 1.24 (1.19-1.29) |
| Anticholinergic | Dicyclomine | 5 | B | 1.19 (1.04-1.37) |
| Antidiabetic | Insulin aspart, human | 6 | B | 1.15 (1.02-1.31) |
| **Benzodiazepine** | | | | |
|  | Diazepam | 6 | N | 1.15 (1.02-1.30) |
|  | Lorazepam | 18 | N | 1.09 (1.02-1.17) |
| **Bronchodilator** | | | | |
|  | Albuterol | 24 | C | 1.11 (1.04-1.18) |
|  | Epinephrine | 7 | C | 1.13 (1.01-1.26) |
|  | Fluticasone propionate and salmeterol (Advair) | 15 | C | 1.12 (1.03-1.21) |
| Cardiovascular | Heparin | 57 | C | 1.06 (1.02-1.11) |
| Corticosteroid | Methylprednisolone^b^ | 49 | C | 1.23 (1.18-1.28) |
| **Fertility** | | | | |
|  | Chorionic gonadotropin^b^ | 12 | X | 1.34 (1.23-1.46) |
|  | Chorionic gonadotropin (human)^b^ | 12 | X | 1.57 (1.43-1.71) |
|  | Chorionic gonadotropin (recombinant) | 56 | X | 1.08 (1.03-1.12) |
|  | Clomiphene^b^ | 13 | X | 1.32 (1.21-1.44) |
|  | Estradiol (Vivelle)^b^ | 5 | N | 1.41 (1.23-1.61) |
|  | Estradiol | 24 | N | 1.07 (1.00-1.13) |
|  | Follicle-stimulating hormone and luteinizing hormone^b^ | 10 | X | 1.42 (1.29-1.56) |
|  | Follitropin^b^ | 19 | X | 1.30 (1.21-1.40) |
|  | Medroxyprogesterone | 6 | X | 1.12 (1.00-1.26) |
|  | Progesterone (Crinone) | 45 | X | 1.06 (1.01-1.11) |
|  | Progesterone, micronized (Prometrium) | 7 | B | 1.12 (1-1.26) |
| **Pain** | | | | |
|  | Lidocaine and prilocaine (EMLA)^b^ | 23 | B | 1.23 (1.16-1.31) |
|  | Sumatriptan | 6 | C | 1.15 (1.02-1.3) |
| Progestogen | Medroxyprogesterone acetate (Provera) | 6 | X | 1.15 (1.02-1.31) |
| Uterotonic agent | Oxytocin | 18 | N | 1.09 (1.02-1.17) |
|  | | | | |
| **Insignificant (*P≥*.05)** | | | | |
| **Antibiotic** |  |  |  |  |
|  | Ciprofloxacin | 14 | C | 0.97 (0.90-1.05) |
|  | Cephalexin | 16 | B | 0.98 (0.91-1.05) |
|  | Sulfamethoxazole and trimethoprim | 16 | C | 0.98 (0.91-1.06) |
|  | Amoxicillin and clavulanate potassium | 9 | B | 0.97 (0.88-1.08) |
|  | Clindamycin | 11 | B | 0.98 (0.89-1.07) |
|  | Azithromycin | 12 | B | 0.98 (0.90-1.07) |
|  | Metronidazole | 180 | N | 1.00 (0.97-1.02) |
|  | Levofloxacin | 7 | C | 0.98 (0.87-1.09) |
|  | Nitrofurantoin | 42 | B | 1.00 (0.95-1.05) |
| **Anticonvulsant** | | | | |
|  | Lamotrigine | 7 | N | 0.97 (0.87-1.09) |
|  | Gabapentin | 6 | N | 0.98 (0.86-1.10) |
|  | Levetiracetam | 6 | C | 0.98 (0.86-1.11) |
| **Antidepressant** | | | | |
|  | Sertraline | 16 | N | 0.97 (0.90-1.05) |
|  | Fluoxetine | 5 | C | 0.97 (0.85-1.11) |
| **Antidiabetic** | | | | |
|  | Metformin | 15 | N | 1.04 (0.96-1.12) |
|  | Insulin isophane, human | 16 | B | 1.03 (0.95-1.11) |
|  | Insulin aspart | 8 | B | 0.98 (0.88-1.09) |
|  | Insulin glargine | 8 | B | 0.98 (0.88-1.09) |
| **Antiemetic** | | | | |
|  | Metoclopramide | 164 | N | 0.97 (0.95-1.00) |
|  | Doxylamine succinate and pyridoxine hydrochloride | 53 | N | 0.97 (0.93-1.01) |
|  | Ondansetron | 246 | B | 0.99 (0.97-1.01) |
|  | Prochlorperazine | 26 | C | 1.01 (0.96-1.08) |
|  | Promethazine | 41 | C | 1.00 (0.95-1.05) |
| **Antifungal** | | | | |
|  | Fluconazole | 52 | D | 1.03 (0.99-1.08) |
|  | Terconazole | 6 | C | 0.98 (0.86-1.11) |
|  | Miconazole | 7 | C | 0.98 (0.87-1.10) |
| **Antihistamine** | | | | |
|  | Diphenhydramine | 72 | B | 1.03 (1.00-1.07) |
|  | Ranitidine | 52 | B | 0.97 (0.93-1.02) |
|  | Doxylamine | 48 | N | 0.98 (0.94-1.02) |
|  | Famotidine | 26 | B | 0.98 (0.92-1.04) |
|  | Cetirizine | 17 | N | 0.98 (0.91-1.05) |
|  | Loratadine | 26 | N | 1.02 (0.96-1.08) |
|  | Fexofenadine | 7 | C | 0.98 (0.87-1.10) |
|  | Hydroxyzine | 6 | C | 0.98 (0.86-1.11) |
| **Antitussive** | | | | |
|  | Acetaminophen and codeine | 10 | C | 1.08 (0.98-1.18) |
|  | Benzonatate | 5 | C | 0.97 (0.85-1.12) |
| **Antiviral** | | | | |
|  | Valaciclovir | 24 | B | 0.98 (0.92-1.04) |
|  | Atovaquone and Proguanil | 6 | C | 0.97 (0.86-1.10) |
|  | Hydroxychloroquine | 9 | N | 0.98 (0.88-1.08) |
|  | Mefloquine | 5 | B | 0.97 (0.85-1.11) |
| Benzodiazepine | Alprazolam | 8 | D | 1.10 (0.99-1.23) |
| Bronchodilator | Budesonide | 8 | N | 0.97 (0.88-1.08) |
|  | Ephedrine | 5 | C | 0.98 (0.85-1.12) |
| **Cardiovascular** | | | | |
|  | Dalteparin | 14 | N | 1.04 (0.96-1.13) |
|  | Labetalol | 15 | C | 0.97 (0.90-1.05) |
|  | Enoxaparin | 47 | N | 1.01 (0.97-1.06) |
|  | Methyldopa | 6 | C | 0.97 (0.86-1.10) |
|  | Amlodipine | 6 | C | 0.97 (0.86-1.10) |
| **Corticosteroid** | | | | |
|  | Prednisone | 12 | D | 1.06 (0.97-1.16) |
|  | Fluticasone | 16 | N | 0.98 (0.91-1.05) |
|  | Hydrocortisone | 9 | C | 0.98 (0.88-1.08) |
|  | Fluticasone propionate | 8 | C | 0.98 (0.88-1.09) |
| Diuretic | Hydrochlorothiazide | 7 | B | 0.98 (0.87-1.10) |
| Expectorant | Guaifenesin | 5 | C | 0.98 (0.85-1.12) |
| **Fertility** | | | | |
|  | Progesterone | 6 | B | 0.90 (0.80-1.02) |
|  | Estradiol (Estrace) | 7 | N | 1.08 (0.97-1.21) |
|  | Cabergoline | 10 | B | 1.05 (0.95-1.15) |
|  | Letrozole | 29 | N | 1.01 (0.95-1.07) |
| **Laxative** | | | | |
|  | Docusate | 93 | N | 1.02 (0.99-1.05) |
|  | Polyethylene glycols | 20 | C | 0.98 (0.91-1.05) |
|  | Bisacodyl | 17 | N | 0.98 (0.91-1.05) |
|  | Magnesium hydroxide | 11 | N | 0.98 (0.89-1.07) |
|  | Sennosides United States Pharmacopeia | 11 | N | 0.98 (0.89-1.07) |
|  | Polyethylene glycols 3350 | 7 | N | 0.98 (0.87-1.10) |
| **Pain** | | | | |
|  | Hydromorphone | 52 | N | 1.03 (0.99-1.08) |
|  | Acetaminophen | 36 | B | 1.03 (0.98-1.09) |
|  | Ibuprofen | 38 | N | 1.03 (0.98-1.08) |
|  | Morphine | 50 | N | 1.02 (0.97-1.06) |
|  | Fentanyl | 26 | C | 0.98 (0.92-1.04) |
|  | Menthol | 21 | N | 0.98 (0.91-1.04) |
|  | Ketorolac | 22 | D | 0.98 (0.92-1.04) |
|  | Benzocaine | 20 | C | 0.98 (0.91-1.05) |
|  | Aspirin | 14 | N | 0.98 (0.90-1.06) |
|  | Naproxen | 22 | N | 1.02 (0.95-1.09) |
|  | Oxycodone | 9 | N | 0.97 (0.88-1.08) |
|  | Acetaminophen and oxycodone hydrochloride | 59 | C | 1.01 (0.97-1.05) |
|  | Meperidine | 6 | N | 0.98 (0.86-1.10) |
| **Progestogen** | | | | |
|  | Medroxyprogesterone acetate | 12 | X | 0.98 (0.90-1.07) |
|  | Ethinyl estradiol and etonogestrel | 9 | N | 0.98 (0.88-1.08) |
|  | Norelgestromin and ethinyl estradiol | 8 | X | 0.98 (0.88-1.09) |
|  | Levonorgestrel | 5 | N | 0.98 (0.85-1.12) |
| **Proton pump inhibitor** | | | | |
|  | Omeprazole | 16 | N | 0.98 (0.91-1.05) |
|  | Esomeprazole | 8 | N | 0.97 (0.88-1.08) |
|  | Lansoprazole | 7 | N | 0.97 (0.87-1.09) |
| Skeletal muscle relaxer | Cyclobenzaprine | 21 | B | 1.06 (0.99-1.14) |
| **Supplement** | | | | |
|  | Sodium chloride | 68 | N | 1.03 (1.00-1.07) |
|  | Pyridoxine | 74 | A | 0.98 (0.95-1.01) |
|  | Folic acid | 49 | A | 0.98 (0.93-1.02) |
|  | Ferrous sulfate | 10 | N | 0.96 (0.87-1.06) |
|  | Vitamin B_12_ | 27 | N | 0.98 (0.92-1.04) |
|  | Magnesium oxide | 17 | N | 0.98 (0.91-1.05) |
|  | Vitamin D | 7 | N | 0.97 (0.87-1.09) |
|  | Calcium carbonate, folic acid, pyridoxine, and vitamin B12 | 13 | N | 0.98 (0.90-1.06) |
|  | Calcium carbonate | 5 | N | 0.97 (0.85-1.11) |
| Thyroid | Levothyroxine | 35 | N | 0.99 (0.94-1.04) |
| **Uterotonic agent** | | | | |
|  | Misoprostol | 7 | X | 0.98 (0.87-1.10) |
|  | Carboprost tromethamine | 5 | C | 0.98 (0.85-1.12) |

^a^US Federal Drug Agency’s 5 letter risk categories (since 2015 replaced with pregnancy and lactation labeling) to indicate the potential of a drug to cause birth defects if used during pregnancy: A (adequate evidence to support safety), B (no adequate studies in humans and animal reproduction studies have failed to demonstrate risk), C (animal reproduction studies show adverse effects and no adequate studies in humans), D (positive evidence of adverse risk on the fetus in humans), X (contraindicated, studies in animals or humans well demonstrate birth defect risk), and N (not formally assigned a category).

^b^*P*<.05 with Bonferroni adjustment.
