# Supplementary material for: Medication-Wide Association Study Using Electronic Health Record Data of Prescription Medication Exposure and Multifetal Pregnancies: Retrospective Study
Source: JMIR Med Inform. 2022 Jun 7;10(6):e32229. doi: 10.2196/32229 (PMC9214620; doi:10.2196/32229)
Supplement: Multimedia Appendix 7 [file medinform_v10i6e32229_app7.docx]

**Appendix 7. Performance analysis confusion tables.**

| **Model 1: No Adjustment** | | | | |
| --- | --- | --- | --- | --- |
| *P*-value (*P* < .05) | MWAS predicted | | | |
|  |  | Positive | Negative | Total |
| Actual-ART | Positive | 12 | 3 | 15 |
|  | Negative | 17 | 91 | 108 |
|  | Total | 29 | 94 |  |
| *P*-value with Bonferroni adj. |  |  |  |  |
| Actual-ART | Positive | 7 | 8 | 15 |
|  | Negative | 4 | 104 | 108 |
|  | Total | 11 | 108 |  |
| **Model 2: Adjustment for Maternal Age** | | | | |
| *P*-value (*P* < .05) | MWAS predicted | | | |
|  |  | Positive | Negative | Total |
| Actual-ART | Positive | 11 | 4 | 15 |
|  | Negative | 16 | 92 | 108 |
|  | Total | 27 | 96 |  |
| *P*-value with Bonferroni adj. |  |  |  |  |
| Actual-ART | Positive | 7 | 8 | 15 |
|  | Negative | 4 | 104 | 108 |
|  | Total | 11 | 112 |  |
| **Model 3: Adjustment for Maternal Age & ART diagnosis/Infertility Diagnosis** | | | | |
| *P*-value (*P* < .05) | MWAS predicted | | | |
|  |  | Positive | Negative | Total |
| Actual-ART | Positive | 11 | 4 | 15 |
|  | Negative | 15 | 93 | 108 |
|  | Total | 26 | 97 |  |
| *P*-value with Bonferroni adj. |  |  |  |  |
| Actual-ART | Positive | 6 | 9 | 15 |
|  | Negative | 4 | 104 | 108 |
|  | Total | 10 | 113 |  |
