# Supplementary material for: Medication-Wide Association Study Using Electronic Health Record Data of Prescription Medication Exposure and Multifetal Pregnancies: Retrospective Study
Source: JMIR Med Inform. 2022 Jun 7;10(6):e32229. doi: 10.2196/32229 (PMC9214620; doi:10.2196/32229)
Supplement: Multimedia Appendix 8 [file medinform_v10i6e32229_app8.pdf]

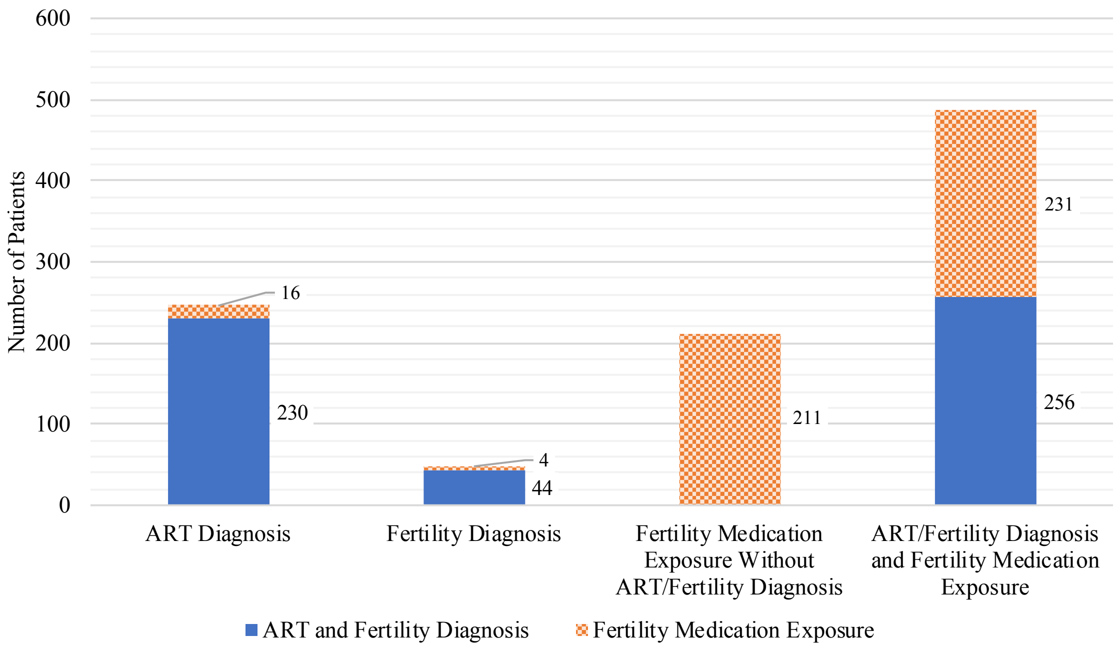

Appendix 8. Stacked Bar Chart of Patients with Assisted Reproductive Technology and Fertility Diagnosis and Patients with Fertility Medication Prescriptions. Assisted Reproductive Technology (ART) and infertility pharmacological treatment associated with multiple birth were defined by the Society for Assisted Reproductive Technology (SART) consumer information and practice guidelines [31]. Patients with pregnancy resulting from ART-use and infertility diagnoses were determined by ICD codes (see **Appendix 2**).
